# Estrogen improves the development of yak (*Bos grunniens*) oocytes by targeting cumulus expansion and levels of oocyte-secreted factors during *in vitro* maturation

**DOI:** 10.1371/journal.pone.0239151

**Published:** 2020-09-17

**Authors:** Yangyang Pan, Meng Wang, Libin Wang, Qian Zhang, Abdul Rasheed Baloch, Honghong He, Gengquan Xu, Jamila Soomro, Yan Cui, Sijiu Yu

**Affiliations:** 1 Gansu Province Livestock Embryo Engineering Research Center, College of Veterinary Medicine, Gansu Agricultural University, Lanzhou, China; 2 National Center for International Research in Cell and Gene Therapy, Sino-British Research Centre for Molecular Oncology, Academy of Medical Sciences, Zhengzhou University, Zhengzhou, China; 3 Department of Veterinary Physiology and Biochemistry, Faculty of Animal Husbandry and Veterinary Sciences, Sindh Agriculture University, Tandojam, Pakistan; Peking University Third Hospital, CHINA

## Abstract

The estrogen-signalling pathway is critical for normal follicular development; however, little is known about its importance during *in vitro* maturation (IVM) in large animals, particularly yaks (*Bos grunniens*). Through the present study, we aimed to determine the mechanisms underlying estrogen involvement in cumulus expansion and the subsequent development of cumulus-oocyte complexes (COCs). COCs were cultured in the maturation medium supplemented with different concentrations (10^−6^–10^−3^ mM) of 17β-estradiol (E2) or its receptor antagonist, fulvestrant, and quantitative reverse-transcription polymerase chain reaction (qRT-PCR) and western blot were performed to determine the expression of cumulus-expansion related factors and oocyte-secreted factors (OSFs). The cumulus expansion of COCs was observed using an inverted microscope, and COCs developmental ability were judged by the evaluation of cleavage and blastulation rates per inseminated oocytes by IVF, and the number of cells in the blastocyst. Cumulus expansion increased with 10^−6^–10^−3^ mM E2, but decreased with fulvestrant. *HAS2*, *PTGS2*, *PTX3* and OSFs expression increased in the 10^−6^–10^−3^ mM E2 groups. Significantly higher cleavage and blastocyst rates were observed in the 10^−4^ mM E2 group than in the fulvestrant and 0 mM E2 groups. Moreover, in the 10^−4^ mM group, blastocysts at 7 days had higher cell counts than the other groups. In conclusion, the increase in cumulus expansion and subsequent oocyte development after the addition of E2 to IVM medium may have resulted from increased cumulus-expansion-related factor expression and OSF levels.

## Introduction

The cumulus expansion of mammalian cumulus-oocyte complexes (COCs) is an important step towards the normal ovulation that mainly occurs during oocyte maturation [1, 2], and is mediated by hormones and growth factors. Once this process is successfully completed, it has potential effects on subsequent fertilization as well as the embryonic development potential of oocytes [3]. Additionally, to optimize assisted reproductive technologies (ARTs) in mammals, the morphology of cumulus expansion can be potentially helpful as a non-invasive oocyte competence marker [4]. Recent studies support the evidence of the significant correlation between fibroblast growth factor 10 (FGF10) and the extent of cumulus expansion in bovines [5, 6]. In addition to this growth factor, reproductive hormones such as FSH, estrogen and prostaglandin E2 (PGE2), can also induce cumulus expansion and meiosis resumption in mammals [7–10], however, not much is known about their roles in large livestock, especially in the yaks (*Bos grunniens*). When 17β-estradiol (E2) is used in mammalian *in vitro* maturation (IVM) protocols and E2 supplementation (1 μg/mL), IVM medium was found to be effective for meiosis resumption in rat COCs [11]. IVM protocols supplemented with E2 from yak COCs have also been used in our previous studies that referenced IVM protocols in other bovines [12, 13], but the underlying mechanisms of COC maturation in yaks are poorly understood.

The transforming growth factor-β (TGF-β) family is a large group of structurally related cell-regulatory proteins, and among them, FGF10, growth differentiation factor 9 (GDF9) and bone morphogenetic protein 15 (BMP15) are secreted by oocytes and are termed “oocyte-secreted factors” (OSFs). These intrinsic factors bind to type I and type II membrane receptors and are involved in oocyte maturation and cumulus expansion [1, 14, 15]. In addition to the effects of OSFs, the expression of transcripts that encode prostaglandin (PG)-endoperoxide synthase 2 (*PTGS2*), hyaluronan synthase 2 (*HAS2*) and tumour necrosis factor alpha-induced protein 6 (*TNFAIP6*) is essential for normal cumulus expansion. Furthermore, if *PTGS2*, *pentraxin 3 (PTX3)*, or *TNFAIP6* are genetically deleted and/or HAS2 is silenced by RNA interference, it results in the defective cumulus expansion [10, 16]. Two of the main FGF10 receptors are modulated by FSH in bovine oocytes and cumulus cells (CCs) [17], and strong coordination exists among GDF9, E2 and BMP15 for the development of CCs in mice [10]. In sheep ovaries, estrogen receptor-1 (ESR1) and estrogen receptor-2 (ESR2) are involved in estrogen production and can be combined with cytochrome P450 family 19 (Cyp19), which is positively correlated with GDF9, BMP15 and BMP7 levels in large follicles [18]. These data indicate that there is strong crosstalk among reproductive hormones, OSFs, and cumulus expansion, but the effects of reproductive hormones on the expression of these factors in yaks are still unclear. We hypothesize that adding E2 to the IVM medium of yak COCs would promote their development, and determining levels of cumulus-expansion-related factors and OSFs should clarify whether there is a regulatory relationship among estrogen, cumulus expansion and OSFs, which would facilitate ART optimization in yaks.

## Materials and methods

All the media and chemicals used in the present study were bought from the Sigma-Aldrich Chemical Company Co. (St. Louis, MO, USA).

### Ethics statement

The study was approved by the Animal Ethics Committee of Gansu Agricultural University. All experiments were performed in accordance with the relevant guidelines and regulations.

### COCs collection

Ovaries were immediately collected from post-pubertal yaks at the local slaughterhouse (Linxia, Gansu, China) and transferred to the laboratory less than 3 h after the animals were slaughtered. The ovaries were kept in a thermos containing saline (0.9% NaCl) at a temperature of 30–35°C. A 1% antifungal agent was added, and a saline solution was used to wash the ovaries three times at 30–35°C. Aspiration was performed using a 20-mL syringe and 18- to 21-gauge needles for follicles of between 2 and 10 mm in diameter. The selection of oocytes was based on the presence of a compact cumulus investment and homogeneous cytoplasm.

### COCs maturation

The COCs were washed three times with tissue culture medium 199 (TCM-199) supplemented with 3% foetal bovine serum (FBS; Medicorp, Montreal, QC, Canada) before being placed in humidified air (5% CO_2_) at 37°C for 2 h to equilibrate the pH. The maturation medium consisted of TCM-199 plus 10% FBS and 200 mM pyruvate, 50 mg/mL of gentamicin, 100 μg/mL of FSH (Gonal-f; Serono Canada Inc., Ontario, Canada) and different concentrations of E2 (10^−6^ mM, 10^−5^ mM, 10^−4^ mM or 10^−3^ mM) or 2.9 nM fulvestrant. Fulvestrant (Selleckchem, Houston, TX, USA) is an estrogen receptor antagonist. A 400-μL maturation medium was used to grow ~50 COCs in four-well dishes (Nunc, Roskilde, Denmark). The cells were supplemented with 200 μL of oil overlay for 24 h at 37°C in humidified air (5% CO_2_). Mature COCs were divided into two sets in each treatment group: the first set was used to evaluate cumulus expansion and determine the expression of cumulus-expansion-related factors and OSFs, and the remaining COCs were subjected to *in vitro* fertilisation (IVF) to assess their subsequent development.

### Cumulus expansion measurement

**Fig 1:**
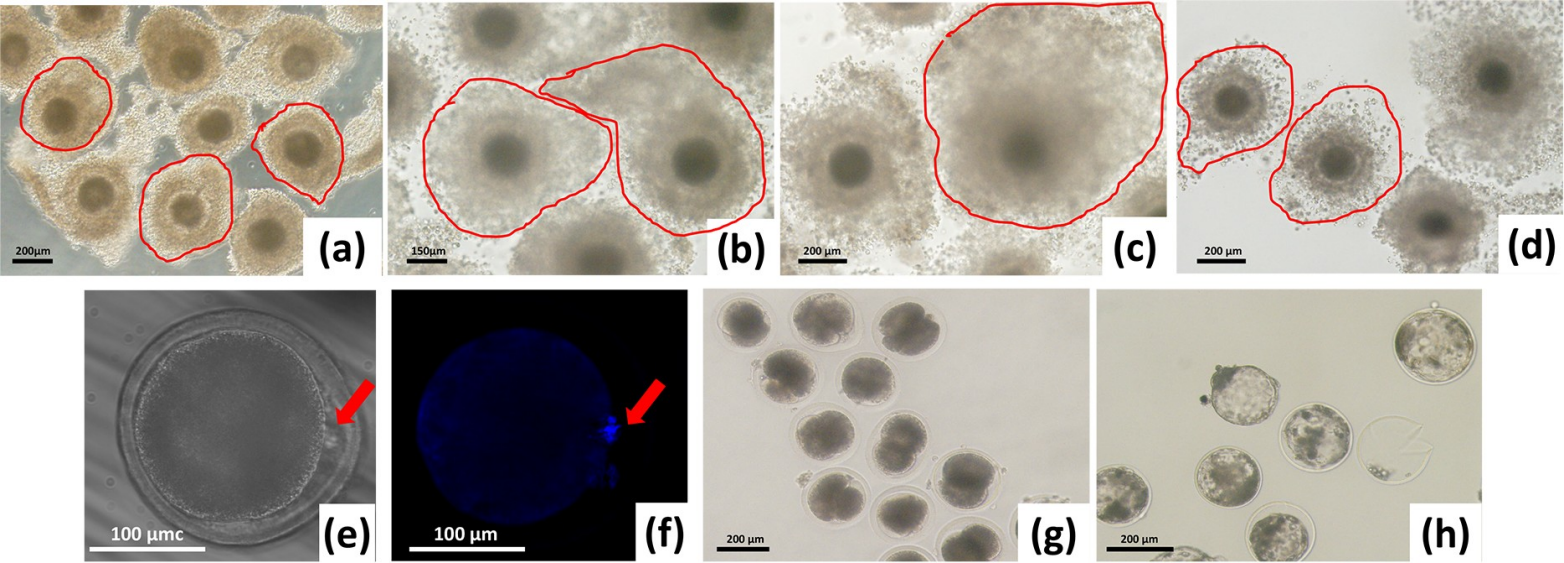
Development of yak cumulus-oocyte complexes (COCs) and embryos produced *in vitro* at different stages, which were used to calculate cumulus expansion, oocyte maturation and embryonic development rates. (a): Immature COCs; (b): Mature COCs in a group without 17β-estradiol (E2); (c): Mature COCs in the 10^−4^ mM E2 group; (d): Mature COCs in the fulvestrant group; (e) and (f): Mature oocytes and stained with DAPI; the arrows indicate the first polar body, which was used to evaluate oocyte maturation in the different treatment groups; (g): Cleaved embryo; (h): Blastocyst. Size of Bar was noted in each figure.

Cumulus expansion was measured following the methods described by Diógenes et al. [5, 6]. Briefly, measurement was done for each COC after selection and then maturation by using ImageJ software (http://imagej.nih.gov/ij). The cumulus expansion after measurement was performed to check the difference between the mean area of all COCs in each group before and after *in vitro* maturation to determine the IVM (Fig 1).

### Detection of cumulus-expansion-related factors and OSFs in mature COCs

#### Detection of cumulus-expansion-related factor and OSF mRNA by quantitative reverse-transcription polymerase chain reaction (qRT-PCR)

Three replicates were taken from each sample for total RNA extraction (RNeasy Micro Kit; Qiagen, Valencia, CA, USA). The first strand was synthesized in a final volume of 20 μL using a Superscript III First Strand Synthesis Kit (Invitrogen, Chicago, IL, USA). The synthesized cDNA was stored in a freezer (-20°C) for downstream applications.

GDF9, FGF10 and BMP15 were selected because they are important OSFs, and genes that are involved in cumulus expansion were also selected [3, 5, 6, 19]. We used qRT-PCR (ABI ViiA 7; Applied Biosystems, Foster City, CA, USA) to calculate the relative gene abundance. Specific primer sequences, amplicon sizes, annealing temperatures and primer concentrations are given in Table 1. In each qPCR, we replicated samples from the same cDNA source three times in order to ensure the reproducibility of the results. For each primer, melting point curves were constructed after amplification to validate the qPCR product identity. Gel electrophoresis on standard 2% agarose gel stained with ethidium bromide was used to confirm amplicon size after the PCR, and the loaded gels were visualized under ultraviolet light. We determined the Ct values of *β-Actin* (a housekeeping gene) in each treatment and found a coefficient of variation of 1.04%, with an average of 28.73 ± 0.48. The relative abundance of each gene was determined using the −ΔΔCt method, with efficiency correction [20]. The results were first normalised to the transcript level of *β-Actin* (ACTB) based on results from bovines, and yaks in particular [6]. Results are expressed as transcript levels relative to those in the control group. Each reaction was conducted in at least three independent experiments that were conducted in duplicate.

**Table 1:** Primer sequences and cycling conditions used in real-time PCR.

Gene	Sequence 5’ to 3’	Temperature (°C)	Fragment size (bp)	Genbank accession no.
*GDF9*	CAAATGGATTGAGATTGATGTG	58°C	194	NM_174681.2
	GAGCACTTGTGTCGTTCAGATA			
*FGF10*	GAAGGGGAAACTCTATGGCTCG	61°C	220	NM_001206326.1
	CTATGAGTGTACCACCATCGGAA			
*BMP15*	GGCACATACAGACCCTGGACTT	60°C	112	NM_001031752.1
	GAGAGGTGGGAATGAGTTAGGTG			
*HAS2*	TCTTTCTCATTGCCACGGTAA	59°C	242	NM_174079.3
	GTAAACCAAACGGATACGGGA			
*PTGS2*	CACCATTTGGCTACGGGAA	60°C	295	NM_174445.2
	TGTTAAACTCAGCAGCAATACGG			
*PTX3*	GCTATCGGTCCATAATGCTTG	59°C	147	NM_001076259.2
	CCGAGTCACCATTTACCCACA			
*TNFAIP6*	CAAAGGAGTGTGGTGGTGTGTT	60°C	188	NM_001007813.2
	TTCAACATAGTCAGCCAAGCAA			
*β-actin*	CTTCAACACCCCTGCCAT CTCGGCTGTGGTGGTGAAG	60°C	238	JF_830811.1

#### Detection of cumulus-expansion-related factors and OSFs by western blot

Based on the effects of E2 on cumulus expansion and maturation of yak oocytes, COCs in groups without E2, with 10^−4^ mM E2 and with 2.9 nM fulvestrant were selected to investigate the effects of E2 on HAS2, PTGS2, PTX3, TNFAIP6, GDF9, FGF10 and BMP15 protein levels. Radioimmunoprecipitation assay buffer supplemented with 1 mM phenylmethylsulfonyl fluoride (Solarbio, Beijing, China) was used to lyse the COCs for 30 min at 4°C. The lysed samples were then centrifuged at 10,000 × *g* (15 min, 4°C) and the lysate (supernatant) was collected to analyse the protein concentration. Protein was divided into equal amounts and then denatured at 100°C before being resolved by sodium dodecyl sulphate-polyacrylamide gel electrophoresis. The samples were then electrotransferred into polyvinylidene fluoride membranes (Bio-Rad Laboratories, Hercules, CA, USA) before western blotting was performed using anti-HAS2 (1:200, ab131364; Abcam), PTGS2 (1:500, ab23672; Abcam), PTX3 (1:400, ab94649; Abcam), TNFAIP6 (1:800, ab128266; Abcam), GDF9 (1:1000, ab93892; Abcam), FGF10 (1:500, ab227102; Abcam) and BMP15 (1:200, ab173886; Abcam) as the primary antibodies. Anti-rabbit immunoglobulin G or anti-goat immunoglobulin G peroxidase was used to identify conjugates and antibody-antigen complexes using an enhanced chemiluminescent detection kit (Beyotime, Nanjing China). A densitometric analysis system (Bio-Rad) was used to determine band intensity under the same conditions, which were normalised to those of *β-Actin* bands.

### Evaluation of subsequent COC development

#### *In vitro* fertilization

The frozen semen from the same yak was provided by the Centre of Livestock Reproductive and Developmental in Qinghai province of China, either from a single straw and/or pooled from a number of straws was used to *in vitro* fertilization. Sperm concentration was determined using a haemocytometer before freezing, and these sperms were used in all experiments. The swim-up procedure was used to separate the sperms, as described previously [21], with small modifications. Briefly, a water bath at 35–38°C was used to thaw the sperm for 60 s; thereafter, the semen was transferred to a 15-mL Falcon tube with 10 mL of Tyrode’s bicarbonate-buffered medium for sperm culture (Sp-TALP) [22]. Capacitation was conducted for 1 h at 37.5°C, before the top 3-mL of medium in the tube was transferred to a clean 15-mL conical centrifuge tube. Spermatozoa that were separated by the swim-up procedure were diluted with Sp-TALP medium, washed three times, and centrifuged at 120 × *g* for 5 min. The supernatant was discarded. Finally, spermatozoan motility and concentration were determined. Sperm was diluted at 1–2 × 10^6^ spermatozoa/mL with Sp-TALP for IVF.

The COCs were rinsed twice at ~30 min before IVF, before being transferred to Fert-TALP medium [23] supplemented with 2 μg/mL of heparin and 50 μg/mL of gentamycin. Spermatozoa were co-incubated with mature COCs in Fert-TALP medium for 18–21 h at 37°C in humidified air (5% CO_2_).

#### Evaluation of cleavage and blastulation rates

Vortexing for 3 min in TCM-199 liberated potential zygotes from the surrounding cumulus cells and excess spermatozoa. Modified synthetic oviductal fluid (mSOF) [24] containing 5 mg/mL of bovine serum albumin (BSA) was used to wash the presumptive zygotes four times. The zygotes were divided into groups of 20 in 100-μL droplets of mSOF with 5 mg/mL of BSA, before being covered with paraffin oil in a humidified atmosphere with 5% CO_2_. The culture medium was replaced with fresh medium every 48 h. The osmolarity and pH of the mSOF were 270–280 mosmoles and 7.2–7.3, respectively. Cleavage and blastulation rates were recorded in all groups at 48 and 192 h after fertilization (Fig 1).

#### Evaluation of blastocyst quality in the different treatment groups

In order to assess blastocyst quality at 192 h, the inner cell mass (ICM), the number of trophectoderm (TE) cells, and the total number of cells were recorded. ICM and TE cells were differentially labelled, as described in a previous study [25]. Briefly, 4% of paraformaldehyde in phosphate-buffered saline (PBS) was used to fix the blastocysts, which were supplemented with 0.5% polyvinylpyrrolidone at 4°C for 12 h. The cells were permeabilized with 1% Triton X-100 in PBS for 30 min at room temperature. Subsequently, embryos were blocked in blocking solution (5% BSA in PBS) for 1 h at room temperature, before they were incubated with primary antibodies against CDX2 (ab88129; Abcam). The primary antibody was recognized as bovine CDX2 at 1:100 for 1 h at room temperature. After being washed thrice for 5 min in PBS at room temperature, the embryos were treated with goat anti-rabbit IgG Alexa Fluor 488 (bs-0295GAF488; Bioss, Beijing, China) secondary antibodies (1:400) for 1 h at room temperature. They were then washed three times in PBS at room temperature. A 1 mg/mL solution of 4′,6-diamidino-2-phenylindole(DAPI) in PBS was used for DNA staining for 3 min. Another round of washing was performed for 10 min, before the blastocysts were mounted under coverslips on glass slides. The embryos were observed and photographed using a fluorescence microscope (Leica, Solms, Germany) equipped with a DFC 350 digital camera (Leica). The total number of cells per blastocyst and the numbers of TE and ICM cells were counted. Three replicates were included in each experiment, and within each replicate, five embryos were studied.

### Statistical analysis

Independent triplicates were included in each experiment, while in one experiment technical triplicates were included. A Student’s *t*-test (dependent, two-tailed) was used to test for significant differences in cumulus expansion among the groups, which was normally distributed. Differences in cleavage and blastocyst rates, as well as the total cell number in blastocysts, were evaluated using the chi-squared test. Repeated-measures analyses of variance (ANOVAs) followed by Tukey-Kramer multiple comparison tests were performed to investigate differences in the relative expression levels of mRNA and proteins. All statistical tests were performed using IBM SPSS Statistics (v22.0), and all values are expressed as the mean ± standard error of the mean with significance set at *P* < 0.05.

## Results

### Effects of E2 on cumulus expansion and maturation of yak oocytes

**Fig 2:**
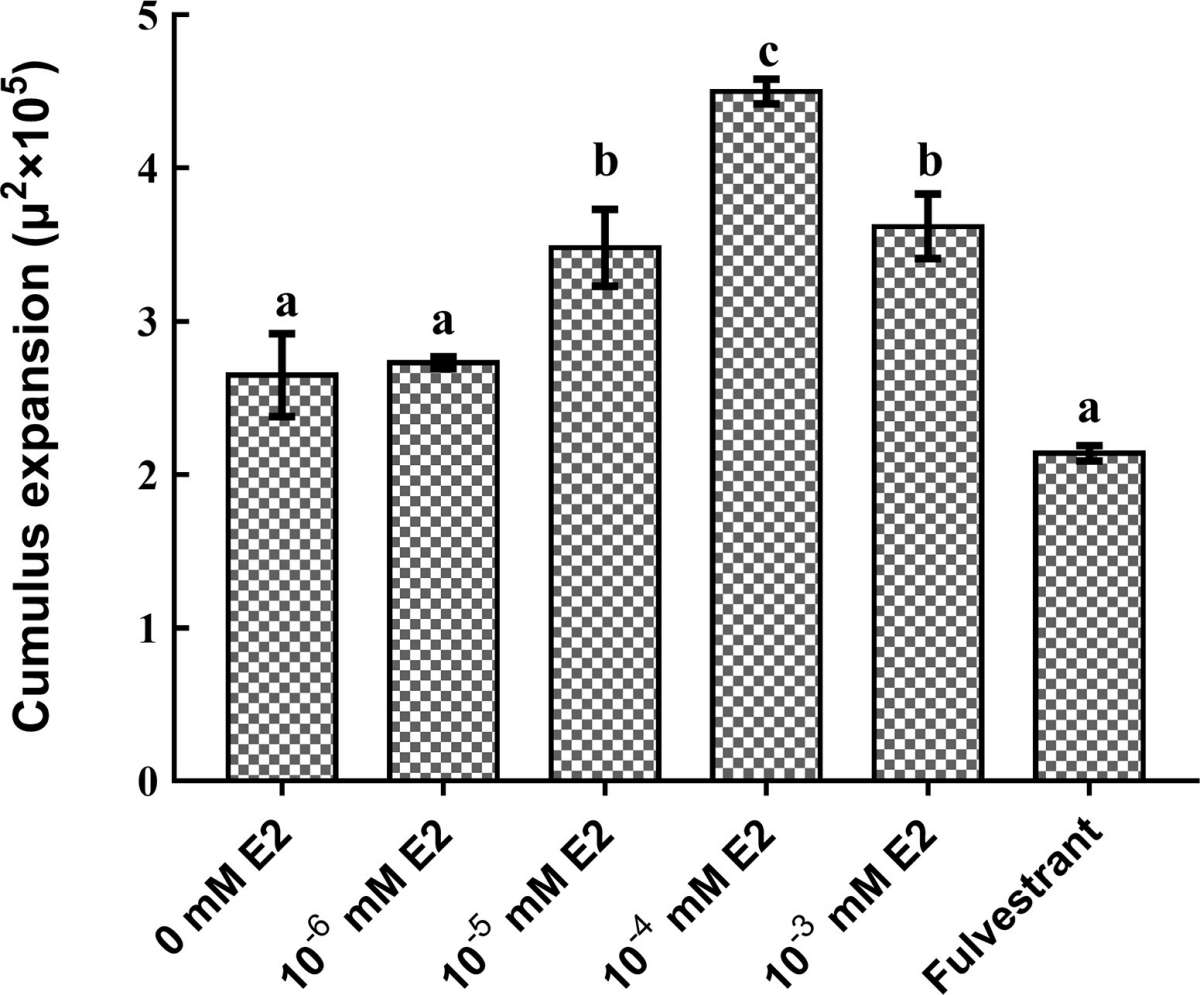
Expansion areas of cumulus–oocyte complexes (COCs) matured for 22 h in media supplemented with different concentrations of 17β-estradiol (E2) or 2.9 nM fulvestrant. Different letters on the bars indicate values that differed significantly (*P* < 0.05).

We investigated the effects of supplementing IVM medium with different concentrations of E2 and 2.9 nM fulvestrant on cumulus expansion. The observation of cumulus expansion of COCs in 10^−5^ mM E2 to 10^−3^ mM E2 groups were similar, which in 10^−4^ mM was shown in Fig 1. The 10^−5^ mM E2 and 10^−3^ mM E2 groups had significantly larger COC expansion areas than the control group and the groups with other E2 concentrations, and there was no difference among 10^−5^ mM E2 to 10^−3^ mM E2 groups (Fig 2). The COC expansion area was small in the group with 2.9 nM fulvestrant (Fig 1), but there was no significant difference with the control group (Figs 1 and 2), both of them were higher than in immature COCs (Fig 1).

We investigated E2 effects on yak oocyte maturation after CC dissociation at 24 h after maturation (Fig 1). The maturation rate was significantly higher when cultured with E2, and was highest in the 10^−4^ mM E2 group. The maturation rate in the fulvestrant group (68.28±0.49%) was significantly lower (Table 2).

**Table 2:** Effects of E2 on maturation of yak oocytes.

Groups	No. of immature COCs	No. of mature COCs	Maturation rate (%)
0 mM E2	443	325	73.31±0.58^a^
10^−6^ mM E2	442	315	74.71±0.69^b^
10^−5^ mM E2	428	347	80.93±0.24^c^
10^−4^ mM E2	470	397	84.39±0.79^d^
10^−3^ mM E2	427	343	80.48±0.51^c^
Fulvestrant	415	283	68.28±0.49^e^

Data were presented as the mean ± standard error of the mean (n = 5).

^a,b,c,d,e^The superscript letters within a row for a particular parameter differ significantly.

### Effects of E2 on *GDF9*, *FGF10* and *BMP15* expression in mature COCs

**Fig 3:**
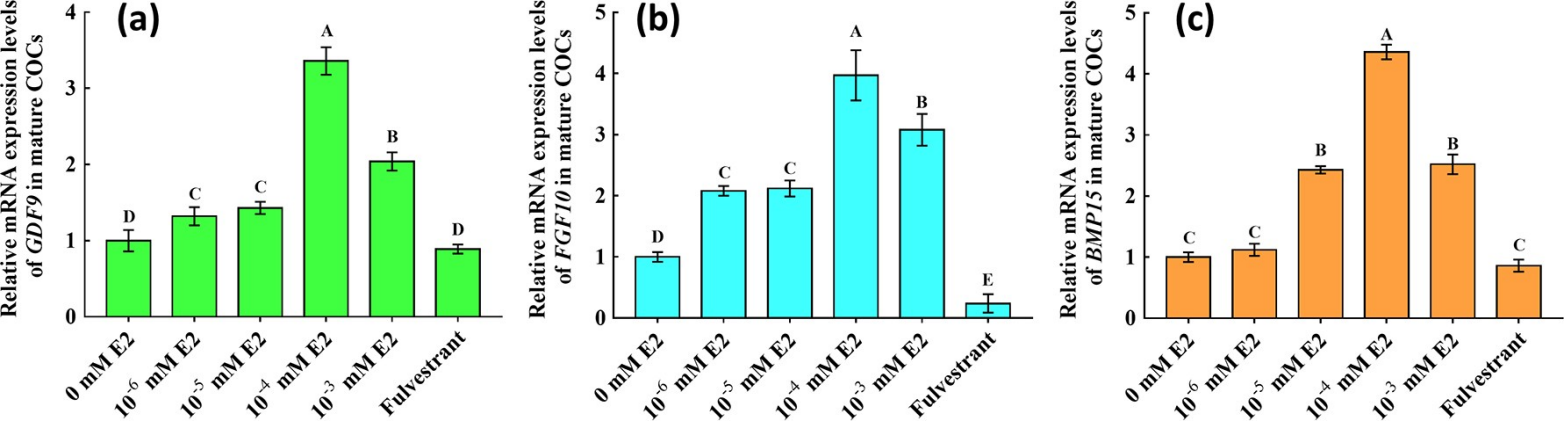
Relative mRNA expression of *GDF9*, *FGF10* and *BMP15* in mature cumulus-oocyte complexes (COCs). Expression levels are shown as relative quantities and were analysed using real-time polymerase chain reaction. *β-Actin* was used to normalise each gene, and mature COCs without 17β-estradiol **(**E2) were used as calibrators. (a), (b) and (c) represent the relative levels of *GDF9*, *FGF10* and *BMP15* mRNA in mature COCs. Significant differences between different groups are indicated by different letters on the bars (*P* < 0.05).

**Fig 4:**
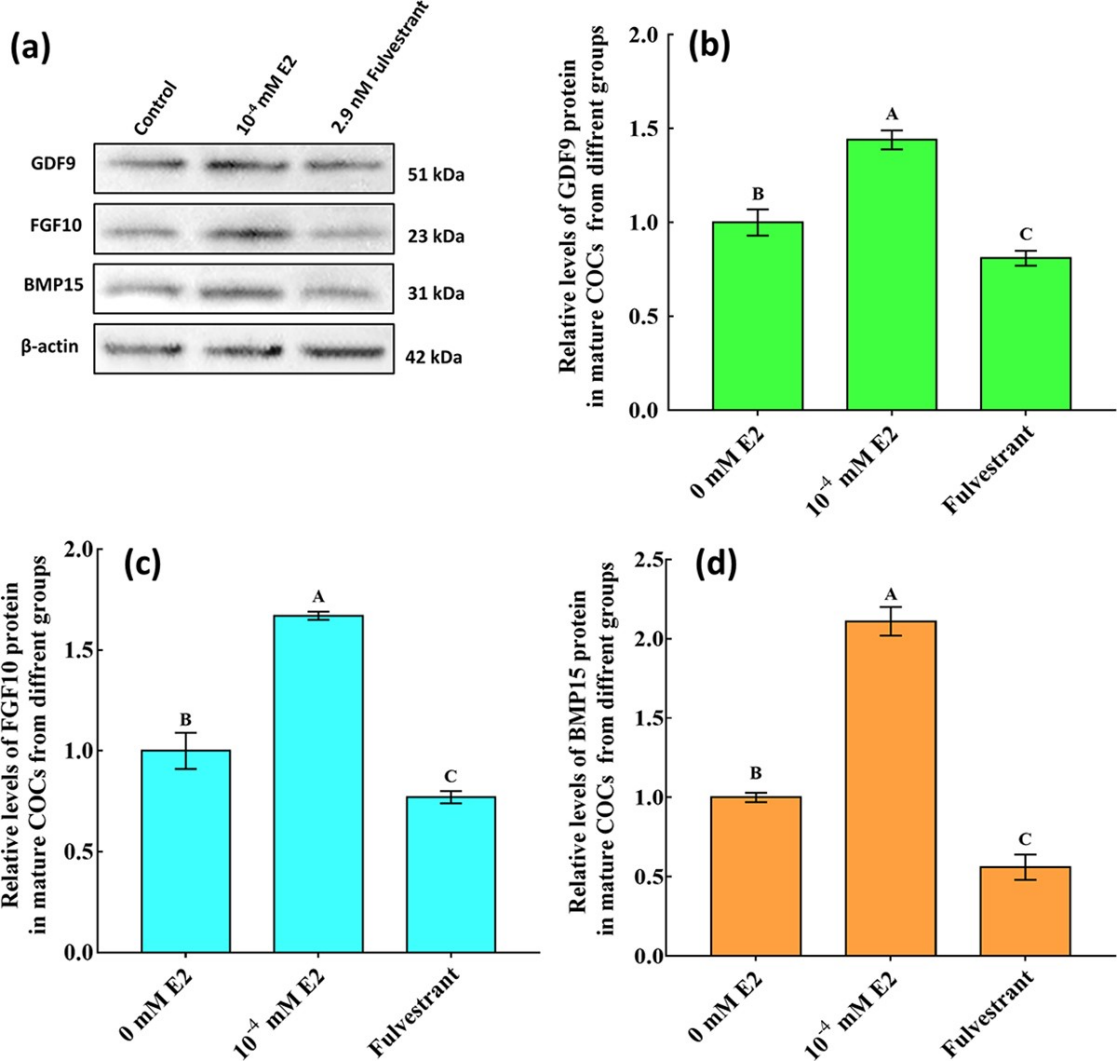
Relative abundances of GDF9, FGF10 and BMP15 proteins in mature cumulus-oocyte complexes (COCs). (a): Expression levels of GDF9, FGF10 and BMP15 proteins as detected by western blotting in mature COCs in different treatment groups. (b), (c) and (d) represent the relative abundances of GDF9, FGF10 and BMP15 proteins in mature COCs, respectively. Expression levels are shown as relative quantities. *β-Actin* was used to normalise each protein, and mature COCs without 17β-estradiol (E2) were used as calibrators. Significant differences between different groups are indicated by different letters on the bars (*P* < 0.05).

E2 significantly increased *GDF9*, *FGF10* and *BMP15* transcription, with the highest levels in the 10^−4^ mM E2 group (*P* < 0.05; Fig 3). Based on obtained results of GDF9, FGF10, and BMP15 transcripts in COCs from the different groups, the corresponding protein levels were determined in COCs from the groups without E2, and with 10^−4^ mM E2, 2.9 nM fulvestrant (Fig 4). Levels of GDF9, FGF10 and BMP15 proteins in both COCs had significantly increased in the 10^−4^ mM E2 group, whereas they had significantly decreased in the fulvestrant group (Fig 4).

### Effects of E2 on the expression of cumulus-expansion-related factors in mature COCs

**Fig 5:**
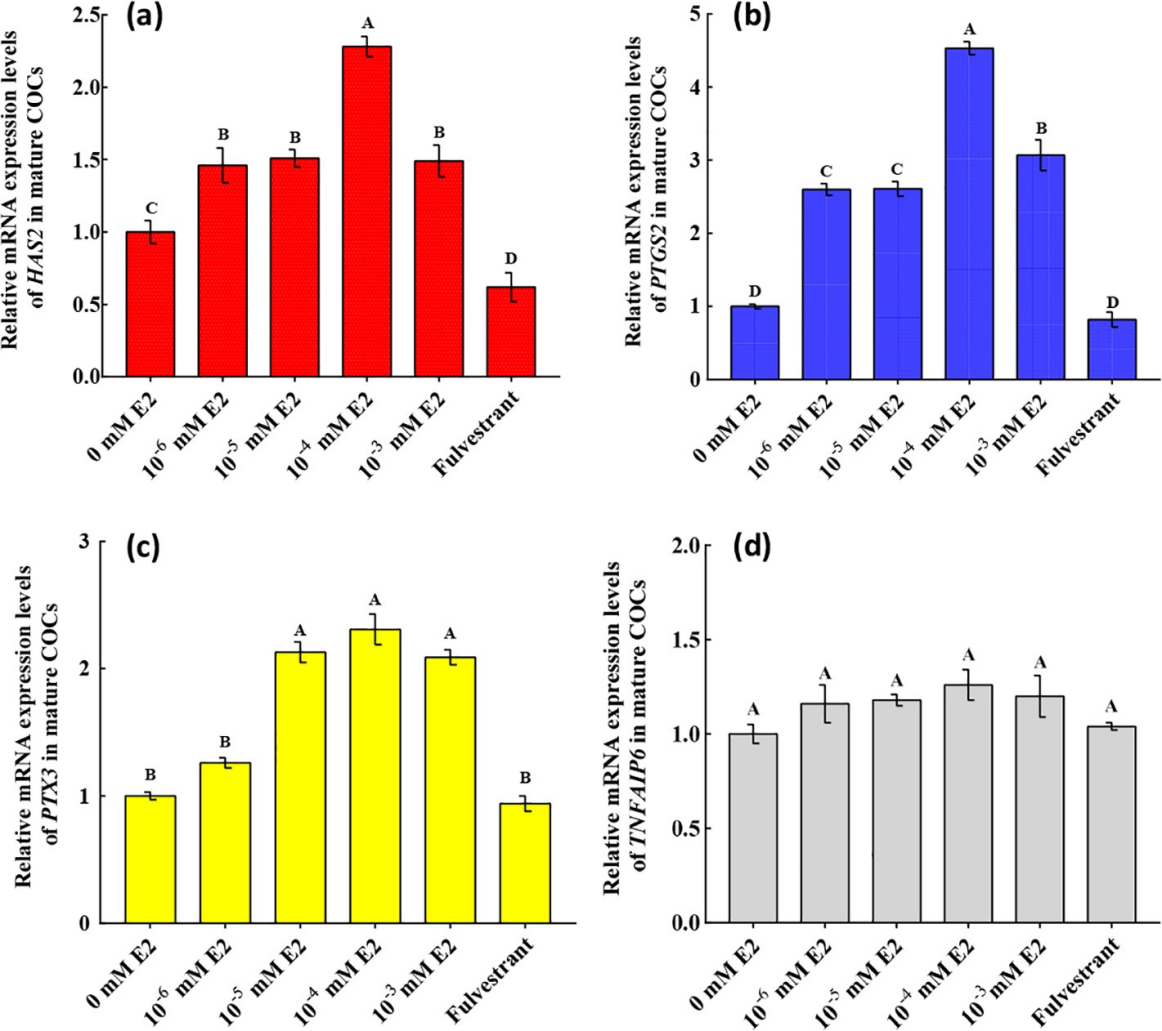
Relative mRNA expression levels of *HAS2*, *PTGS2*, *PTX3* and *TNFAIP6* in mature cumulus-oocyte complexes (COCs). Expression levels are shown as relative quantities and were analysed using real-time polymerase chain reaction. *β-Actin* was used to normalise gene expression, and mature COCs without 17β-estradiol (E2) were used as calibrators. (a), (b), (c) and (d) represent the relative levels of *HAS2*, *PTGS2*, *PTX3*, and *TNFAIP6* mRNA in mature COCs, respectively. Significant differences between different groups are indicated by different letters on the bars (*P* < 0.05).

**Fig 6:**
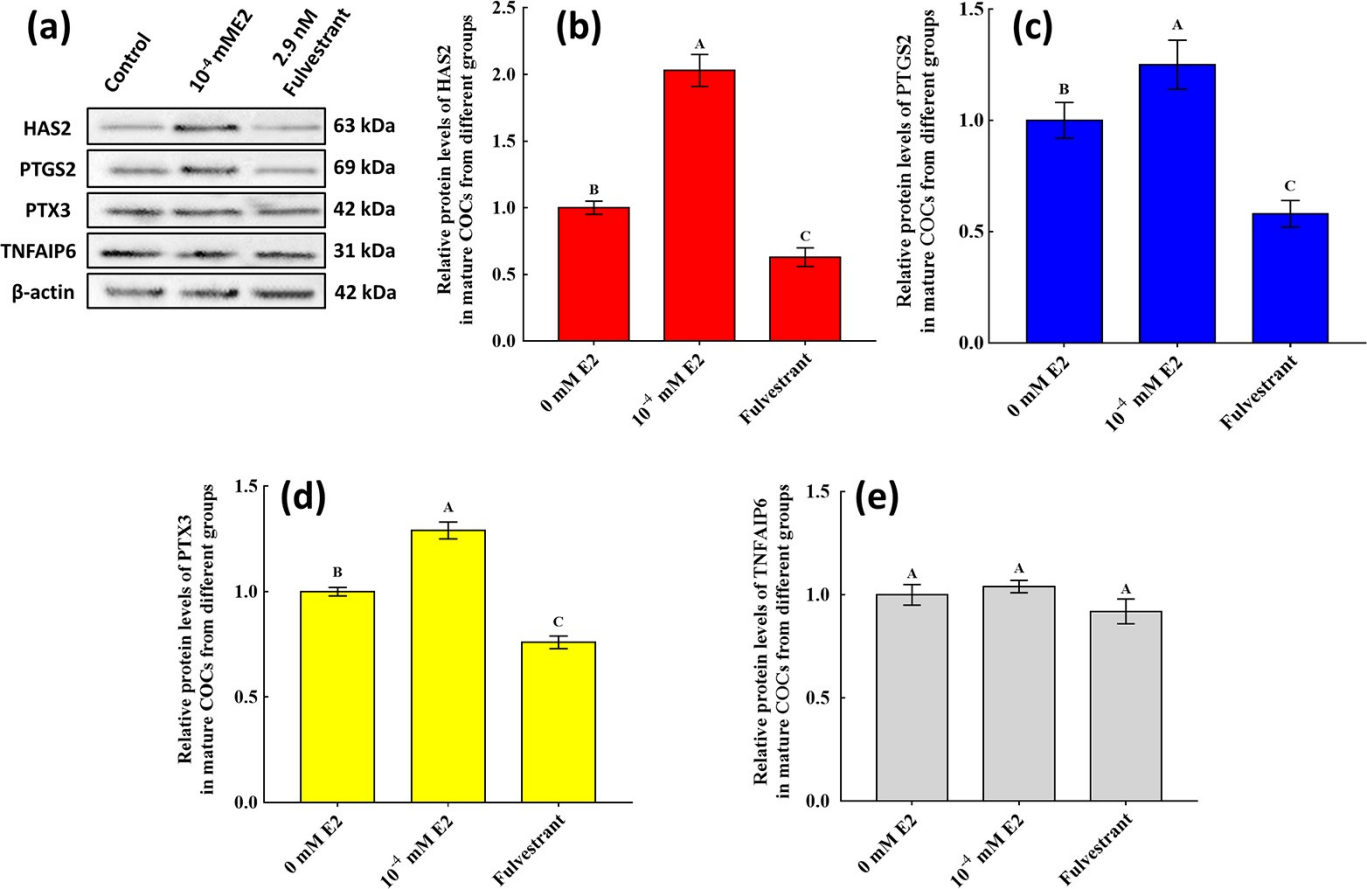
Relative abundances of HAS2, PTGS2, PTX3 and TNFAIP6 proteins in mature cumulus-oocyte complexes (COCs). (a): Expression levels of HAS2, PTGS2, PTX3 and TNFAIP6 proteins as determined by western blot in mature COCs in different treatment groups. (b), (c), (d) and (e) represent the relative abundances of HAS2, PTGS2, PTX3, and TNFAIP6 proteins in mature COCs, respectively. Expression levels are shown as relative quantities. *β-Actin* was used to normalise each protein, and mature COCs without 17β-estradiol (E2) were used as calibrators. Significant differences between different groups are indicated by different letters on the bars (*P* < 0.05).

In order to investigate the molecular profile of the E2 effect on cumulus expansion, we analysed changes in the expression of cumulus-expansion-related genes in COCs in the different groups, which revealed that transcript levels of *HAS2*, *PTGS2* and *PTX3* were significantly increased by E2 (*P* < 0.05; Fig 5). The greatest changes in gene expression were observed in the group treated with 10^−4^ mM E2 (Fig 5). *TNFAIP6* expression was unaffected by E2 (Fig 6). Protein changes were similar to the changes in their corresponding transcript levels, and there were significant increases in HAS2, PTGS2 and PTX3 with 10^−4^ mM E2 (*P* < 0.05; Fig 6). TNFAIP6 protein levels did not differ significantly among the groups.

### Effects of E2 on the subsequent development of mature COCs

The effects of E2 on the developmental competence of fertilized embryos in the treatment groups are presented in Table 3. The 10^−4^ mM E2 group during IVM had cleavage and blastocyst rates of 77.37±1.24% and 20.53±1.28%, respectively, which were significantly higher than those of the control group. Cleavage and blastocyst rates decreased in the fulvestrant group (Table 3).

**Table 3:** Developmental competence of yak IVF embryos from different groups.

Groups	No. of mature COCs	No. of cleaved embryos (%)	NO. of Blastocysts (%)
0 mM E2	283	194(69.40±1.38^a^)	40(14.11±1.36^a^)
10^−4^ mM E2	317	245(77.37±1.24^b^)	65(20.53±1.28^b^)
Fulvestrant	343	225(65.53±1.68^c^)	38(11.17±1.02^c^)

The superscript letters (a, b, c) within a column for a particular parameter differs significantly.

The cleavage rate: number of cleavage embryos/number of matured oocytes observed.

The blastocyst rate: number of blastocyst/number of matured oocytes observed.

**Fig 7:**
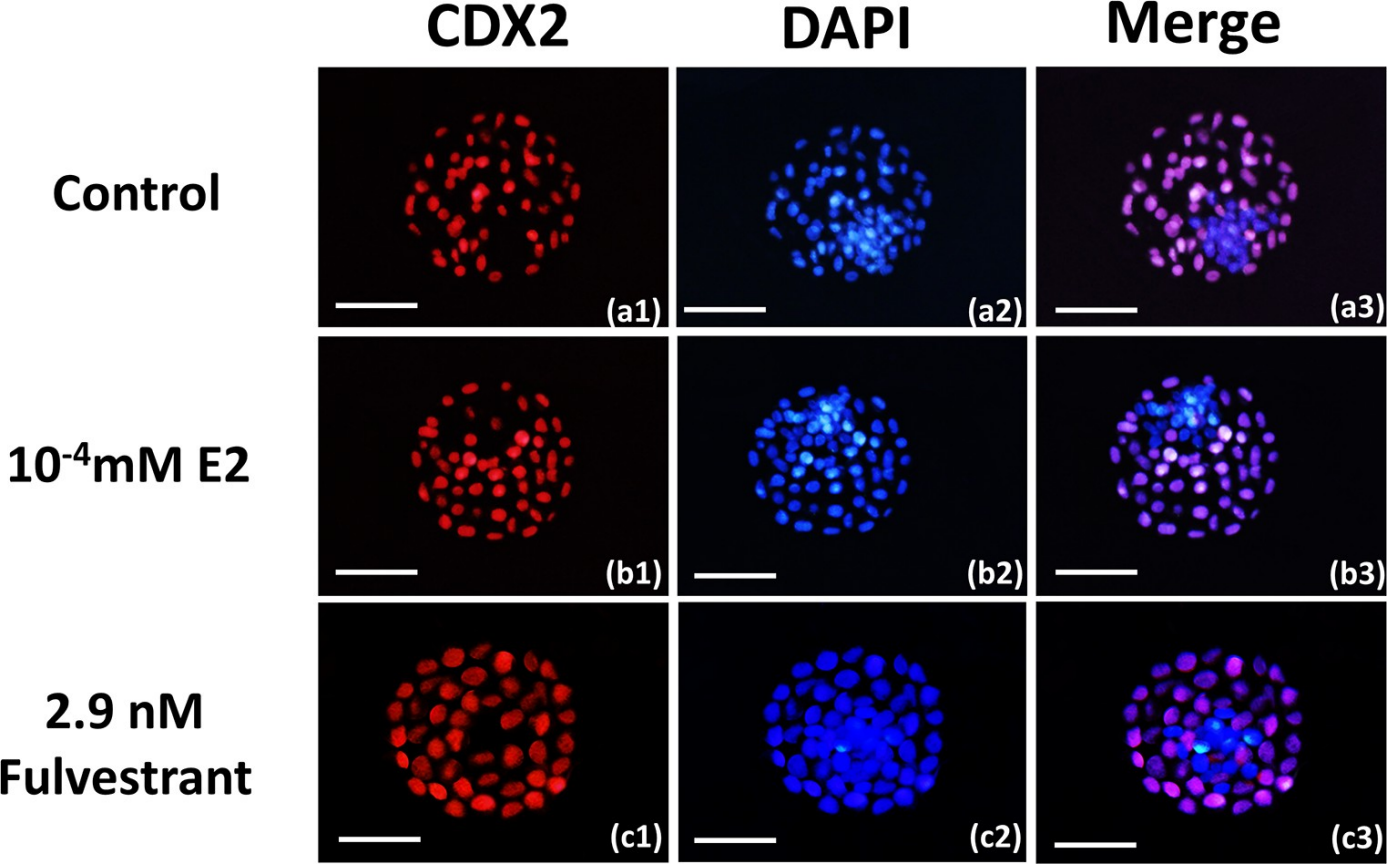
Total cell numbers and their allocation to the inner cell mass (ICM) and trophectoderm (TE) lineages of yak blastocysts in different treatment groups. TE cells stained with CDX2 appear red, and total cell nuclei stained with 4′,6-diamidino-2-phenylindole appear blue. Bar = 100 μm in panels.

Blastocysts that were produced *in vitro* after 8 days of culture were subjected to differential cell staining for cell allocation analysis (Fig 7, Table 4). The mean total cell number in the blastocysts of the 10^−4^ mM E2 group during IVM was significantly higher than that of the other groups (*P* < 0.05; Fig 7, Table 4). In addition, the numbers of ICM and TE cells in the blastocysts of the 10^−4^ mM E2 group during IVM were significantly higher than those of the other groups (*P* < 0.05; Fig 7, Table 4). However, the ratio of the number of TE cells to the total number of cells in the blastocysts did not significantly differ among the groups (*P >* 0.05; Fig 7, Table 4).

**Table 4:** Total cell numbers and their allocation to the ICM and TE lineages of yak blastocysts from different groups.

Groups	Total no. of cells	No. of TE cells	No. of ICM cells	TE: Total cell (%)
0 mM E2	108.67^a^	84.67^a^	24.00^a^	77.91±0.86^a^
10^−4^ mM E2	122.67^b^	94.67^d^	28.00^b^	77.16±1.09^a^
Fulvestrant	106.67^a^	83.33^a^	23.33^a^	78.12±0.99^a^

The superscript letters (a, b) within a column for a particular parameter differs significantly.

## Discussion

Yaks are an important source of income for people and also serve as a means of transportation [26, 27]. However, harsh environments and the yak’s seasonally polyoestrous nature lower its reproductive efficiency (40–60%) when compared with other types of livestock [28]. Differences in physiology between yaks and other bovine species have resulted in differences in IVM conditions. Although 1 μg/mL E2 was added to the IVM medium for COC maturation, the optimal E2 concentration is unknown, and whether other concentrations have better effects remains to be investigated. The E2 mechanism during COC maturation is complex, and a synergistic effect between E2 and OSFs has been reported in other species [1, 7, 10]; therefore, further studies should investigate whether there is any regulatory relationship between them. In the present study, we investigated E2 effects on the maturation of oocytes in yaks, and their subsequent developmental ability. We found that the developmental ability of mature COCs had significantly improved in the 10^−4^ mM E2 group, indicating that the optimum concentration of E2 during yak IVM is 10^−4^ mM. Knowledge of this value will improve yak ARTs, which would increase yak production.

Bidirectional communication between CCs and oocytes is necessary for mammalian oocyte cytoplasmic maturation and ultimate developmental competence [29], and reproductive hormones from follicular fluid and factors secreted by oocytes are involved in follicular cell function regulation [30]. However, the complex role that E2 plays during IVM is not fully understood, particularly regarding cumulus expansion in large animals. To elucidate the mechanisms of E2 during IVM, we compared cumulus-expansion-related factor and OSF expression in COCs under different conditions, which to the best of our knowledge, is the first such study on yaks. Cumulus expansion is enhanced by exogenous E2 in mice; however, the mechanisms of action of this reproductive hormone are not fully understood in large animals [10]. In the present study, we provide evidence that the enhancement of cumulus expansion during IVM occurs through the modulation of *HAS2*, *PTGS2* and *PTX3* levels by E2. This result is consistent with those from goats and humans, which indicated that cumulus expansion partly determines the developmental potential of an oocyte [31, 32]. *TNFAIP6* levels were unaffected by exogenous E2 supplementation, which is consistent with results in other bovines and mice [6, 10, 33]. These findings indicated that *TNFAIP6* did not regulate cumulus expansion upon supplementation with exogenous E2. Protein kinase A (PKA) pathway was responsible for *TNFAIP6* promoter activity and transcription i*n vitro* bovine granulosa cell culture [34], which also needed activator protein-1 (AP-1) transcription factor that phosphorylate in extracellular signal-regulated kinase 1/2 (ERK1/2) signalling pathway [35]. The regulation signalling of E2 on PKA during yak COCs maturation remains poorly studied, while these results contribute to the field of knowledge addressing pathway between CCs and oocytes.

Moreover, OSFs are involved in E2 production and in CC response to it [36], which our results support. We found that the exogenous E2 groups had significantly an improvement in COC expansion with an increased expression of GDF9 and BMP15 compared to groups not supplemented with E2 (Figs 2–6). COCs cultured *in vitro* are the closest we can get to the physiological phenomenon of oocyte maturation *in vivo*; therefore, we evaluated the effects of E2 on COCs. Further studies should investigate the underlying mechanism, and the relationship among E2, oocytes and CCs, which need to be cultured separately. In addition, the relationship among estrogen, cumulus expansion and OSFs should be investigated using different experimental strategies. Examples of such an approach include the loss and gain of OSF functions, RNAi depletion, and functional blocking with antibodies. Exogenous E2 can regulate OSF expression, and previous studies have found that OSFs, such as BMP15 and GDF9, enhance bovine oocyte developmental competence and provide evidence that OSF regulation of the COC microenvironment and cumulus expansion are important determinants of oocyte developmental programming [33, 37]. Further studies should use effective OSF inhibitors to confirm that OSF function during E2 induce cumulus expansion in yak COCs.

Apart from investigating E2 effects on cumulus expansion and the maturation of yak oocytes, mature COCs under different conditions were used to perform IVF to evaluate subsequent developmental ability based on cleavage and blastulation rates. We observed significantly higher cleavage and blastulation rates in the exogenous E2 groups than in the control group, indicating that the functions of E2 during IVM affect the subsequent development of IVF embryos. Our findings are in agreement with the effects of FGF10 on oocytes in bovines and colony stimulating factor 2 (CSF2) on yak embryos [6], FGF10 can improve oocyte maturation in vitro by decreasing the percentage of apoptotic oocytes and increasing the relative abundance of developmentally important genes [38]. However, the effects of CSF2 and FGF10 on the developmental capacity of mammal oocytes are significantly weaker than the function of selenium [39], which plays an important role in the proliferation of and steroidogenesis in CCs by activating the adenosine monophosphate-activated protein kinase (AMPK) pathways, thereby increasing the expression of its downstream cell-cycle- and steroid-synthesis-related genes, as well as regulating cellular oxidative stress [40]. E2 effects on the *in vitro* development of goat COCs demonstrate that E2 is able to alter the expression of genes that are related to embryonic development and quality, e.g. connexin 43 (*Cx43*) [41], as well as genes found in the epidermal growth factor receptor (EGFR) and ERK1/2 signalling pathway [42]. Estradiol can induce rapid activation of ERK1/2 and AKT and these signaling events are probably mediated by membrane ER, provide beneficial effects on enhancing the survival and development of embryo and fetus [43]. Therefore, assessing blastocyst quality is crucial for evaluating E2 function. A total cell number close to that in an *in vivo*-derived blastocyst indicates *in vitro* embryonic viability [40], and we found that total cell numbers in the blastocysts were increased in the E2-treated groups during IVM (Table 4, Fig 7). This shows that E2 effects on oocyte maturation and cumulus expansion are critical for the quality of subsequent blastocysts. However, this finding is inconsistent with FGF10 effects in other bovines [6, 17], so further studies should determine whether there are any differences in this regard among species, or whether there are functional differences between E2 and FGF10. A balanced ICM/total cell number ratio is very important for embryonic development, because increased TE cell numbers at the expense of ICM in blastocysts is related to offspring abnormalities [44]. Low ICM values in cloned bovine blastocysts may be one of the reasons for developmental failure, because there may be an insufficient number of cells to sustain further embryonic development after transfer [45]. To the best of our knowledge, this is the first study conducted on the effects of E2 on yak embryos.

## Conclusions

This study has improved our understanding of the effects of E2 on the regulation of yak COC maturation and subsequent development, and has confirmed previous reports that E2, GDF9 and BMP15 act in coordination to promote the development of mammalian cumulus expansion. We found that levels of cumulus-expansion-related factors and OSFs fluctuate under different conditions, suggesting that E2 enhances the subsequent developmental ability of yak oocytes by targeting cumulus expansion and levels of oocyte-secreted factors during IVM.

## Supporting information

S1 File (PDF)
