# Supplementary material for: Estrogen improves the development of yak (Bos grunniens) oocytes by targeting cumulus expansion and levels of oocyte-secreted factors during in vitro maturation
Source: PLoS One. 2020 Sep 17;15(9):e0239151. doi: 10.1371/journal.pone.0239151 (PMC7498018; doi:10.1371/journal.pone.0239151)
Supplement: S1 File — (PDF) [file pone.0239151.s001.pdf]

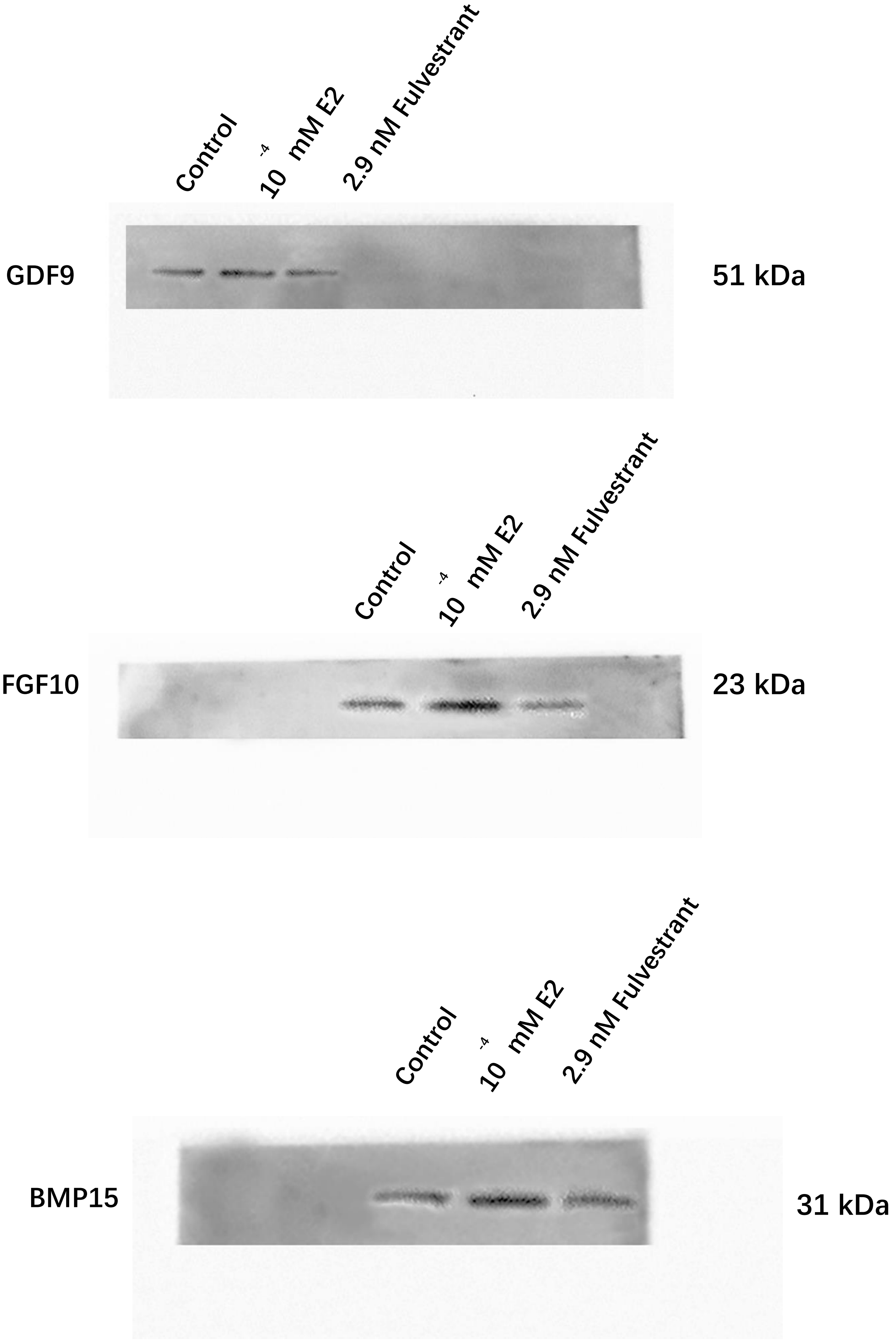
Control
10^{-4} mM E2
2.9 nM Fulvestrant
GDF9
51 kDa
Control
10^{-4} mM E2
2.9 nM Fulvestrant
FGF10
23 kDa
Control
10^{-4} mM E2
2.9 nM Fulvestrant
BMP15
31 kDa

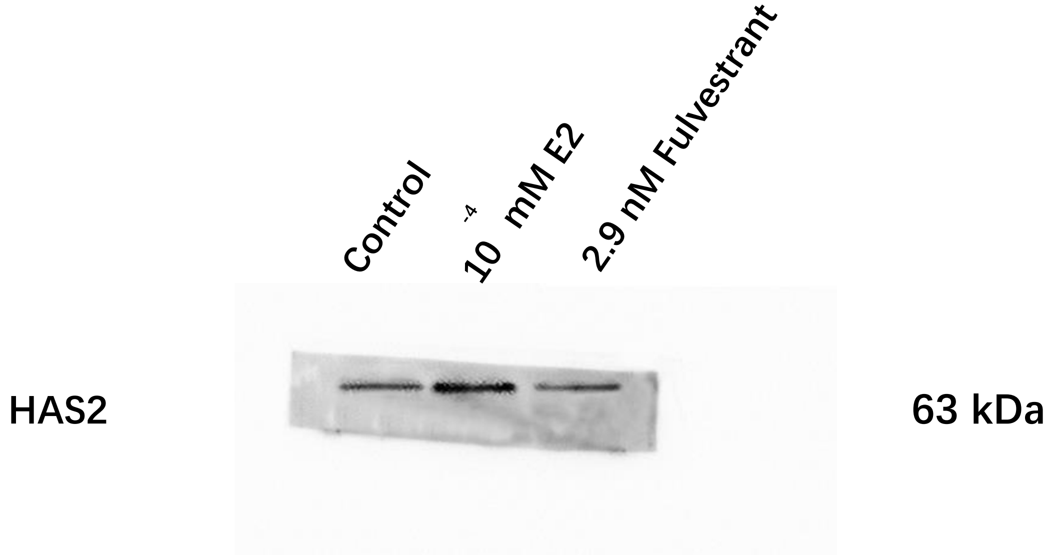
Control
10-4 mM E2
2.9 nM Fulvestrant
HAS2
63 kDa

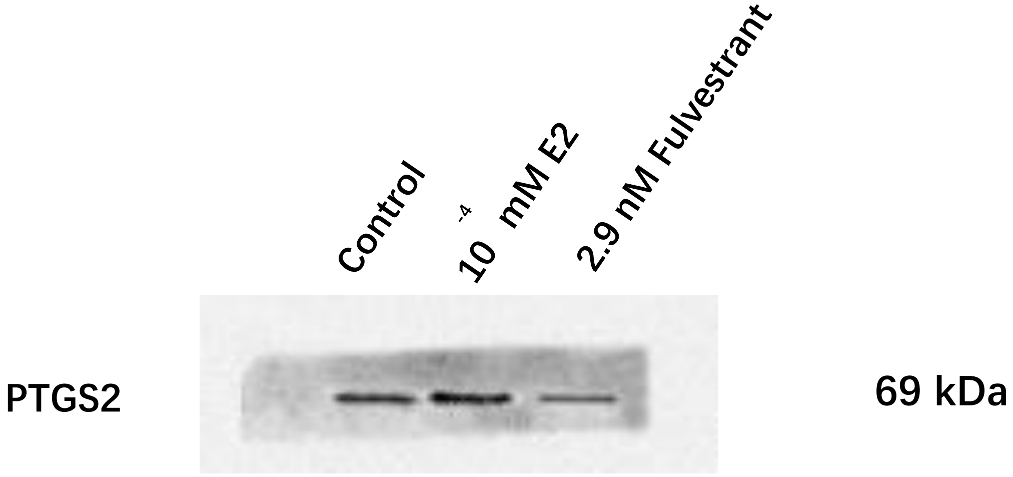
Control
10-4 mM E2
2.9 nM Fulvestrant
PTGS2
69 kDa

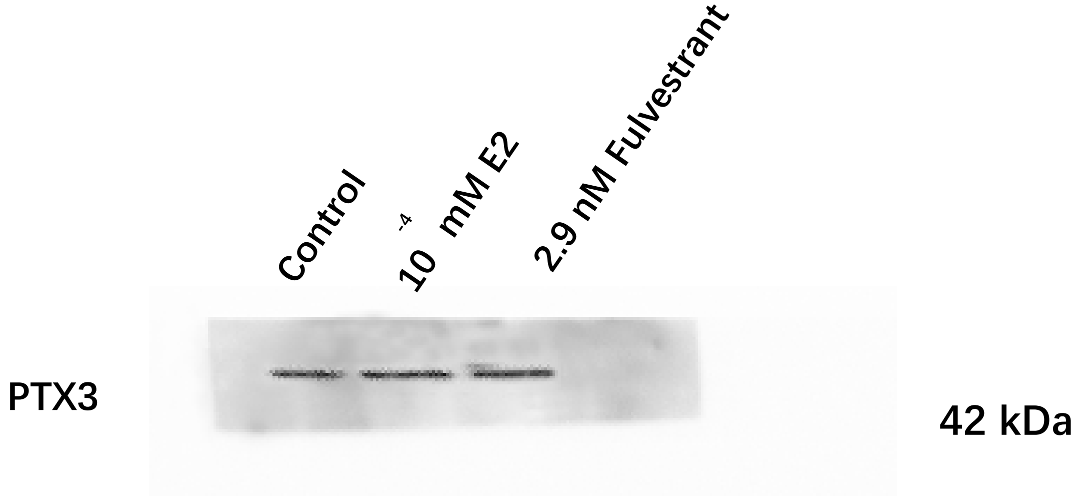
Control
10-4 mM E2
2.9 nM Fulvestrant
PTX3
42 kDa

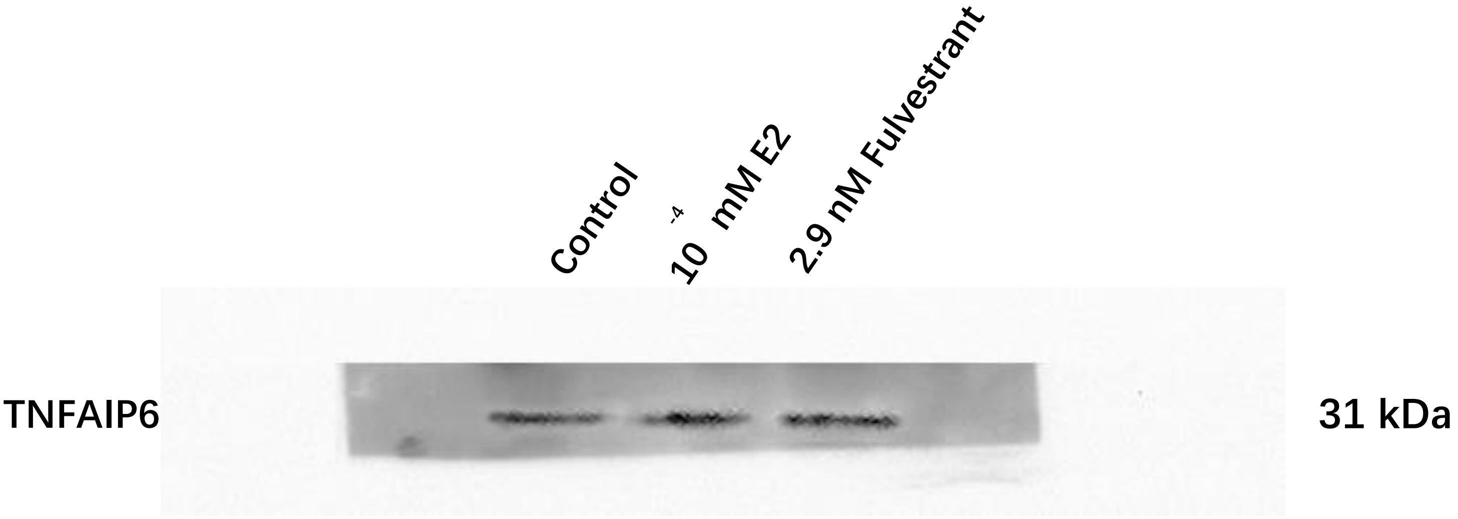
Control
10^{-4} mM E2
2.9 nM Fulvestrant
TNFAIP6
31 kDa

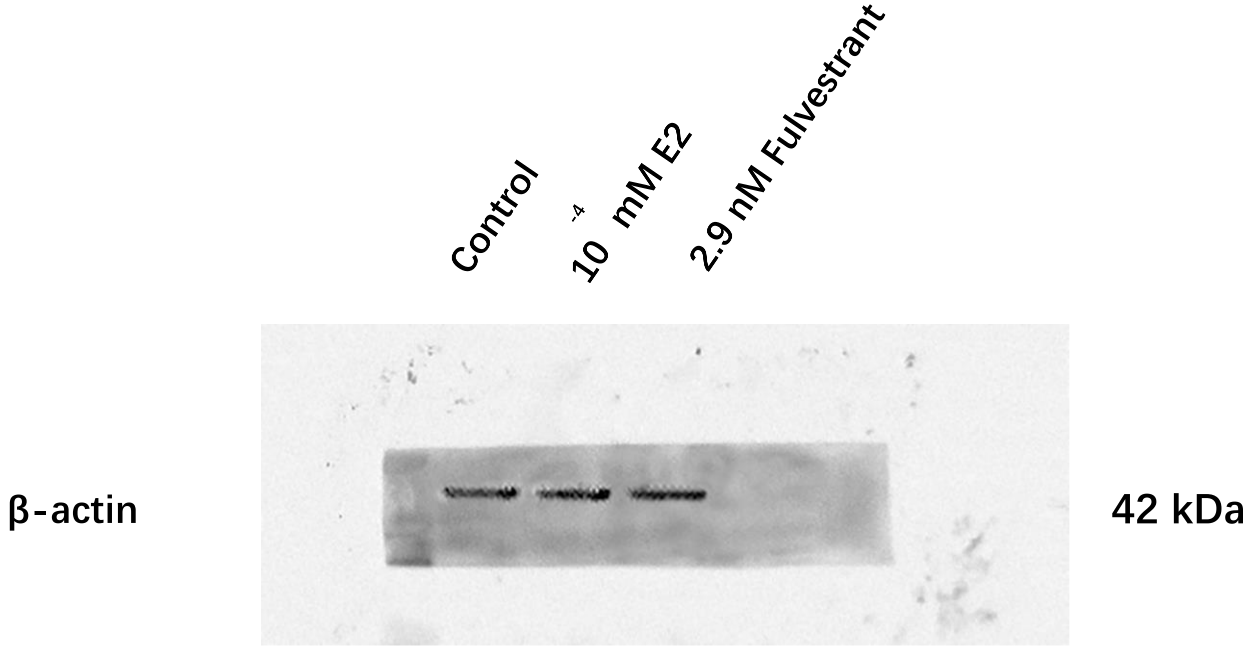
Control
10^{-4} mM E2
2.9 nM Fulvestrant
β-actin
42 kDa
